
JOURNAL INFORMATION
==============================
NLM Title Abbreviation: ScientificWorldJournal
Journal ID: ScientificWorldJournal
NLM Journal ID: 101131163
Journal ID: TSWJ

The Scientific World Journal

ISSN: 2356-6140
EISSN: 1537-744X
Publisher: Wiley

ARTICLE INFORMATION
==============================
PMCID: PMC5974740
PMID: 14755113
DOI: 10.1100/tsw.2003.118
Article ID: 210498
Article version: 1
Subjects: Directions in Science

Syndecan-1 as a Regulator of Chemokine Function

Götte Martin *
Echtermeyer Frank
Departments of Physiological Chemistry and Pathobiochemistry and Obstetrics and Gynecology, University Hospital Münster, D-48149 Münster, Germany
*Martin Götte: mgotte@uni-muenster.de
Publication date: 2003
Electronic publication date: 2003 Dec 11
Volume: 3
First page: 1327
Last page: 1331
Received 2003 Sep 1; Revised 2003 Nov 25; Accepted 2003 Dec 3
Copyright: Copyright © 2003 Martin Götte and Frank Echtermeyer.
Copyright year: 2003
License: This is an open access article distributed under the Creative Commons Attribution License, which permits unrestricted use, distribution, and reproduction in any medium, provided the original work is properly cited.
License URL: https://creativecommons.org/licenses/by/3.0/

Keywords: syndecan, chemokine, heparan sulfate proteoglycan, interleukin, IL-8, KC, CXC, lung fibrosis, leukocyte transmigration, matrix metalloproteinase

Funding: Deutsche Forschungsgemeinschaft SFB492 Interdisciplinary Centre for Clinical Research Protogenia Research Grant

==============================
Directions in Science
*TheScientificWorldJOURNAL* (2003) 3, 1327–1331
ISSN 1537-744X; DOI 10.1100/tsw.2003.118

### Syndecan-1 as a Regulator of Chemokine Function

Martin Götte[§,*] and Frank Echtermeyer

*Departments of Physiological Chemistry and Pathobiochemistry and [§]Obstetrics and Gynecology, University Hospital Münster, D-48149 Münster, Germany*

**E-mails**: mgotte@uni-muenster.de; fechter@uni-muenster.de

*Received September 1, 2003; Revised November 25, 2003; Accepted December 3, 2003; Published December 11, 2003*

---

**KEYWORDS:** syndecan, chemokine, heparan sulfate proteoglycan, interleukin, IL-8, KC, CXC, lung fibrosis, leukocyte transmigration, matrix metalloproteinase

**DOMAINS:** inflammation, lymphokines and chemokines, glycoscience, endothelial function, extracellular matrix, immunology, cell biology, biochemistry, pulmonary pathology

---

Chemokines are a family of chemotactic cytokines that play critical roles in leukocyte recruitment to sites of inflammation. Characterized by the presence of conserved aminoterminal cysteine residues, these low-molecular weight proteins signal through G-protein–coupled receptors with seven transmembrane domains[1,2]. Apart from their function in leukocyte recruitment, chemokines and their receptors have been ascribed roles in angiogenesis and tumor growth, infections, Th1 and Th2 responses, as well as the maturation and development of several leukocyte subpopulations[1]. Considering their fundamental role in orchestrating the inflammatory response, it is no surprise that a dysregulation of chemokine function is observed in a variety of pathological conditions, such as pulmonary fibrosis, hypersensitivity responses, chronic inflammation, and cancer[1,2,3,4]. For this reason, it is important to know how chemokine action is regulated in molecular detail, in order to develop more efficient therapies for diseases characterized by chemokine malfunction.

Two recent papers shed more light on the regulation of chemokine function, uncovering a crucial role for the cell surface heparan sulfate (HS) proteoglycan syndecan-1 in the generation of chemokine gradients[5,6]. Using mice deficient in the matrix-metalloprotease matrilysin in an experimental model of lung fibrosis, Li et al.[5] observed an impaired transepithelial migration of neutrophils, which resulted in protection against the lethal effect of bleomycin-induced lung injury. They found that matrilysin sheds a complex of syndecan-1 and the CXC chemokine KC from the mucosal surface, which serves as a chemotactic gradient for neutrophils. Marshall et al.[6] used an *in vitro* model of transendothelial migration to demonstrate that syndecan-1 forms a chemotactic IL-8 gradient at the endothelial cell surface. An increase of constitutive syndecan-1/IL-8 complex shedding in the presence of a neutralizing antibody to plasminogen activator inhibitor-1 (PAI-1) resulted in an inhibition of transendothelial neutrophil migration.

The syndecans are a family of cell surface heparan sulfate proteoglycans (HSPG), which act as adhesion molecules, modulators of growth factor function, and coreceptors in processes as diverse as morphogenesis, tissue repair, host defense, tumor development, and energy metabolism[7,8]. Protease-mediated cleavage of the intact syndecan ectodomains (“shedding”) converts the cell-surface molecules into soluble effectors[9]. Increasing evidence suggests an important role for the syndecans in the

*Corresponding author.
©2003 with author.

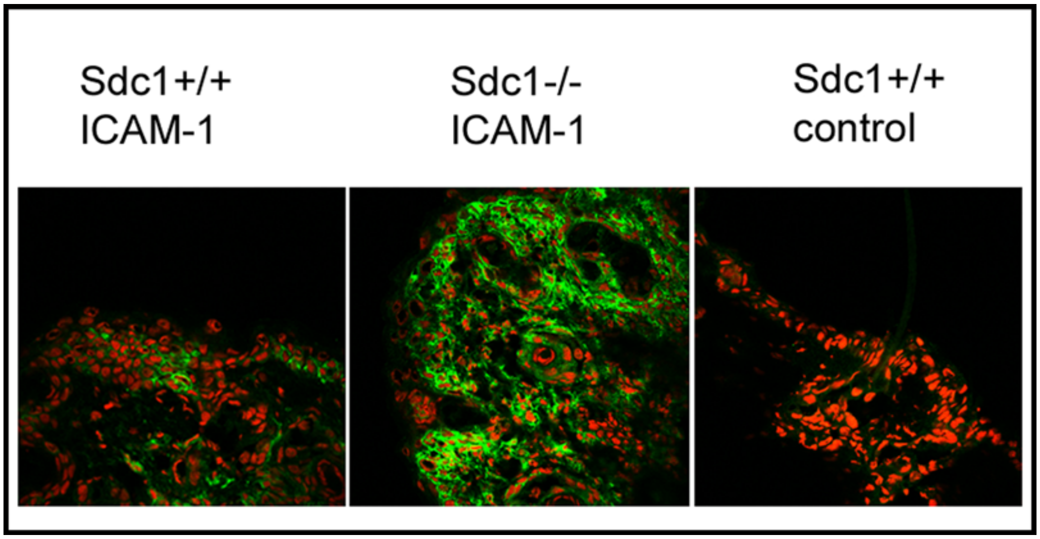

**FIGURE 1.** Increased expression of ICAM-1 in syndecan-1 knockout mice relative to wild-type mice subjected to delayed-type hypersensitivity (DTH). A DTH response to oxazolone was elicited in syndecan-1 wild-type (Sdc1+/+) and knockout (Sdc1–/–) mice[11]. Oxazolone-treated ears were harvested at the peak of the inflammatory response and cryosectioned. After immunostaining with ICAM-1 (left and central panel) or rat IgG isotype control (right panel) primary antibodies, and AlexaFluor488 conjugated goat-antirat secondary antibodies (green staining), the sections were subjected to confocal laser microscopy as described[12]. Nuclei were stained with propidium iodide (red staining).

regulation of inflammation[4]. For example, mice lacking syndecan-1 display increased leukocyte-endothelial interactions and angiogenesis, as well as increased inflammatory responses in experimentally induced delayed-type hypersensitivity[10,11] (Fig. 1). The major functional domains of the syndecans, their heparan sulfate chains, have been implicated in regulating the activity of chemokines for some time[13,14,15]: All chemokines bind to HS through positively charged domains, and for many chemokines a modulation of their activity by HS has been demonstrated (reviewed in [4]). For example, HS binding to IL-8 leads to a structural stabilization of the dimeric form of the chemokine, which results in a prolonged biological activity and enhanced neutrophil responses to IL-8[13,16,17,18]. Most recently, it has been demonstrated that glycosaminoglycan binding and the capability to oligomerize are essential requirements for the activity of a number of chemokines *in vivo*[19].

The study published by Li et al.[5] confirms and extends these findings on the role of HSPGs, and establishes a role for syndecan-1 in the formation of chemokine gradients. Following their initial observation of an increased expression of the matrix-metalloprotease matrilysin (MMP-7) in lungs injured by bleomycin instillation, they subjected matrilysin knockout (MAT–/–) mice to bleomycin-induced lung fibrosis. MAT–/– mice were found to be protected against bleomycin-induced lethality to a surprisingly high extent: At a bleomycin dose that led to lethality in all control mice after 10 days, 80% of the MAT–/– mice were still alive. Apart from the survival benefit, a reduction in alveolar fibrosis was noted in the knockout mice. An impaired ability of neutrophils to migrate from the interstitium into the lumenal compartment was identified as the cause of the reduced mortality of MAT–/– mice. This could clearly be demonstrated by instilling the chemotactic peptide nFNLP along with bleomycin: The forced neutrophil influx restored bleomycin-induced lethality in the MAT–/– mice. The authors were able to unravel the molecular mechanism underlying the neutrophil compartmentalization in the MAT–/– mice when they discovered that the CXC chemokine KC, a functional homologue of human IL-8, was markedly elevated in the bronchoalveolar lavage of bleomycin-treated wild-type mice, but remained at baseline levels in MAT–/– mice. Interestingly, KC protein levels were elevated in total lung extracts, but remained constant in wild-type lung extracts following bleomycin treatment. Thus, KC was present, but could not be released into the alveolar space. In an elegant set of experiments, Li et al. could finally demonstrate that KC can be coimmunoprecipitated with the ectodomain of syndecan-1, and that matrilysin induces the shedding of syndecan-1 from the surface of the lung epithelium. Syndecan-1–/– revealed similar results as

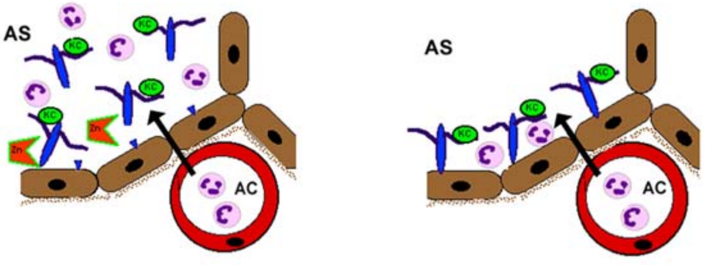

**FIGURE 2.** Generation of a chemokine gradient by syndecan-1 in a model of pulmonary fibrosis (cf. [5]). Left panel: the chemokine KC (green) is bound to syndecan-1 (blue) and shed from the lung epithelium into the alveolar space (AS) on cleavage of the syndecan-1 core protein by matrilysin (red). In bleomycin-treated wild-type, neutrophils extravasate from alveolar capillaries (AC) and follow the KC/syndecan-1 gradient, leading to a massive influx of neutrophils into the alveolar space and ultimately to lung fibrosis. Right panel: in matrilysin-deficient mice, the complex of KC and syndecan-1 remains at the epithelial cell surface. Neutrophils are only found at the epithelium, but not in the alveolar space, resulting in decreased lung fibrosis and mortality

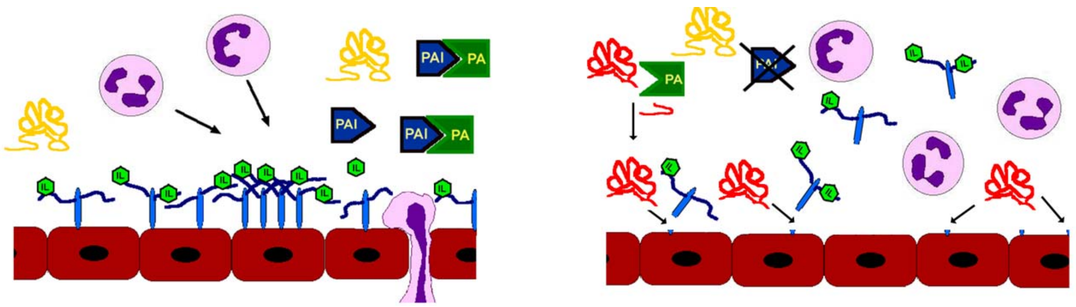

**FIGURE 3.** Generation of a transendothelial interleukin-8 gradient by syndecan-1 (cf. [6]). Left panel: interleukin-8 (green) is bound to syndecan-1 (blue), which forms a gradient at the surface of HUVEC cells. Neutrophils are directed to this chemotactic gradient, resulting in a recruitment to the HUVEC surface and ultimately in transmigration. In the presence of plasminogen activator inhibitor-1 (PAI), plasminogen activators (PA) are kept inactive, and plasminogen (orange) is not activated. Right panel: on inactivation or down-regulation of PAI-1, plasminogen activators generate plasmin (red), which is shedding syndecan-1/IL-8 complexes from the HUVEC surface. The chemotactic gradient for neutrophils is destroyed, resulting in decreased transmigration of neutrophils.

MAT–/– mice in terms of KC levels in lung tissue and bronchoalveolar lavage and a reduction in neutrophil numbers after bleomycin treatment. In summary, the data indicate that matrilysin generates a transepithelial chemotactic gradient by releasing a complex of syndecan-1 and the chemokine KC into the alveolar space (Fig. 2).

A role for syndecan-1 in the generation of a chemokine gradient was also demonstrated by Marshall et al.[6], who observed that the major part of IL-8 was present in the culture media of HUVEC cells, but not cell associated. The authors could show an increased cell association of IL-8 in the presence of the protease inhibitor aprotinin, or PAI-1, respectively. Inhibition of PAI-1 activity with a blocking antibody resulted in increased shedding of syndecan-1, which could be coimmunoprecipitated with IL-8, into the HUVEC culture medium. Interestingly, PAI-1 inhibition did not only increase the release of syndecan-1/IL-8 complexes, it also reduced transendothelial migration of neutrophils in a dose-dependent manner. This effect could be reverted by the plasmin inhibitor aprotinin. The study suggests that shedding of syndecan-1/IL-8 complexes is mediated by plasmin, and that PAI-1 stabilizes the chemoattractant form of IL-8, complexed to syndecan-1, at the endothelial cell surface (Fig. 3).

In summary, a role for syndecan-1 in the generation of chemokine gradients was demonstrated both for neutrophils migrating though an epithelium and through an endothelial cell layer. In both cases, proteases mediated the shedding of chemokine-syndecan-1 complexes, which served as a leukocyte chemoattractant.

However, the consequences of protease-mediated shedding were different: In lung epithelium, a soluble gradient was required to support neutrophil migration, whereas in the case of the endothelium, the syndecan-1/chemokine complex was required at the cell surface. It has been shown that soluble IL-8 has inhibitory effects on inflammation (e.g., neutrophil recruitment), whereas a proinflammatory effect has been ascribed to cell-surface bound IL-8 (discussed in [6]). Thus, neutrophil migration through endothelial cell layers and epithelia appear to have different requirements (cf. [20]). Both studies bring previous, seemingly unrelated data into a functional context: Binding of chemokines to the HS chains of syndecan-1 is not only a means of stabilizing the chemokine in an active, oligomeric form[15,18,19], it also provides a means of selectively regulating the (trans)migration of neutrophils through protease-mediated shedding of the syndecan ectodomain[5,6,9]. These results help to explain the observation of increased leukocyte-endothelial interactions, inflammation, and angiogenesis in a mouse model lacking syndecan-1[10,11]. In addition, they open up new pathways for the treatment of diseases like pulmonary fibrosis, chronic inflammation, and cancer through the development of selective syndecan-shedding inhibitors, and the potential use of syndecan-1, or syndecan-1–neutralizing reagents as therapeutics.

### ACKNOWLEDGMENTS

Original work by the authors was financially supported by the Deutsche Forschungsgemeinschaft (SFB492, project B12), the Interdisciplinary Centre for Clinical Research (IZKF, project D18) (F.E.) and a Protogenia research grant (M.G.).

### REFERENCES

1. Rossi, D. and Zlotnik, A. (2000) The biology of chemokines and their receptors. *Annu. Rev. Immunol.* **18**, 217–242.
2. Johnston, B. and Butcher, E.C. (2002) Chemokines in rapid leukocyte adhesion triggering and migration. *Semin. Immunol.* **14**, 83–92.
3. Mukaida N. (2003) Pathophysiological roles of interleukin-8/CXCL8 in pulmonary diseases*. Am. J. Physiol. Lung Cell Mol. Physiol.* **284**, L566–L577.
4. Götte, M. (2003) Syndecans in inflammation. *FASEB J.* **17**, 575–591.
5. Li, Q., Park, P.W., Wilson, C.L., and Parks, W.C. (2002) Matrilysin shedding of syndecan-1 regulates chemokine mobilization and transepithelial efflux of neutrophils in acute lung injury. *Cell* **111**, 635–646.
6. Marshall, L.J., Ramdin, L.S., Brooks, T., Charlton, P., and Shute, J.K. (2003) Plasminogen activator inhibitor-1 supports IL-8-mediated neutrophil transendothelial migration by inhibition of the constitutive shedding of endothelial IL-8/heparan sulfate/syndecan-1 complexes. *J. Immunol.* **171**, 2057–2065.
7. Bernfield, M., Götte, M., Park, P.W., Reizes, O., Fitzgerald, M.L., Lincecum, J., and Zako, M. (1999) Functions of cell surface heparan sulfate proteoglycans. *Annu. Rev. Biochem.* **68**, 729–777.
8. Echtermeyer, F., Streit, M., Wilcox-Adelman, S., Saoncella, S., Denhez, F., Detmar, M., and Goetinck, P. (2001) Delayed wound repair and impaired angiogenesis in mice lacking syndecan-4. *J. Clin. Invest.* **107**, R9–R14.
9. Fitzgerald, M.L., Wang, Z., Park, P.W., Murphy, G., and Bernfield, M. (2000) Shedding of syndecan-1 and -4 ectodomains is regulated by multiple signaling pathways and mediated by a TIMP-3-sensitive metalloproteinase. *J. Cell Biol.* **148**, 811–824
10. Götte, M., Joussen, A.M., Klein, C., André, P., Wagner, D.D., Hinkes, M.T., Kirchhof, B., Adamis, A.P., and Bernfield, M. (2002) Role of syndecan-1 in leukocyte-endothelial interactions in the ocular vasculature. *Invest. Ophthalmol. Vis. Sci.* **43**, 1135–1141.
11. Götte, M., André, P., Bryce, P.J., O'Reilly, M., Oettgen, H., Wagner, D.D., and Bernfield, M. (2003) Syndecan-1 KO mice exhibit increased inflammatory and delayed-type hypersensitivity responses. *Eur. J. Cell Biol.* **82(Suppl. 53),** 24–25.
12. Schaefer, L., Macakova, K., Raslik, I., Micegova, M., Gröne, H.J., Schönherr, E., Robenek, H., Echtermeyer, F.G., Grässel, S., Bruckner, P., Schaefer, R.M., Iozzo, R.V., and Kresse, H. (2002) Absence of decorin adversely influences tubulointerstitial fibrosis of the obstructed kidney by enhanced apoptosis and increased inflammatory reaction. *Am. J. Pathol.* **160**, 1181–1191.
13. Webb, L.M., Ehrengruber, M.U., and Clark-Lewis, I. (1993) Binding to heparan sulfate or heparin enhances neutrophil responses to interleukin 8. *Proc. Natl. Acad. Sci. U. S. A.* **90**, 7158–7162.
14. Wang, D., Sai, J., and Richmond, A. (2003) Cell surface heparan sulfate participates in CXCL1-induced signaling. *Biochemistry* **42**, 1071–1077.
15. Slimani, H., Charnaux, N., Mbemba, E., Saffar, L., Vassy, R., Vita, C., and Gattegno, L. (2003) Binding of the CC-chemokine RANTES to syndecan-1 and syndecan-4 expressed on HeLa cells. *Glycobiology* **13**, 623–634

16. Frevert, C.W., Kinsella, M.G., Vathanaprida, C., Goodman, R.B., Baskin, D.G., Proudfoot, A., Wells, T.N., Wight, T.N., and Martin, T.R. (2003) Binding of interleukin-8 to heparan sulfate and chondroitin sulfate in lung tissue. *Am. J. Respir. Cell. Mol. Biol*. **28**, 464–472.
17. Spillmann, D., Witt, D., and Lindahl, U. (1998) Defining the interleukin-8-binding domain of heparan sulfate. *J. Biol. Chem.* **273**, 15487–15493.
18. Goger, B., Halden, Y., Rek, A., Mosl, R., Pye, D., Gallagher, J., and Kungl, A.J. (2002) Different affinities of glycosaminoglycan oligosaccharides for monomeric and dimeric interleukin-8: a model for chemokine regulation at inflammatory sites. *Biochemistry* **41**, 1640–1646.
19. Proudfoot, A.E., Handel, T.M., Johnson, Z., Lau, E.K., LiWang, P., Clark-Lewis, I., Borlat, F., Wells, T.N., and Kosco-Vilbois, M.H. (2003) Glycosaminoglycan binding and oligomerization are essential for the in vivo activity of certain chemokines. *Proc. Natl. Acad. Sci. U. S. A.* **100**, 1885–1890.
20. Colgan, S.P., Comerford, K.M., and Lawrence, D.W. (2002) Epithelial cell-neutrophil interactions in the alimentary tract: a complex dialog in mucosal surveillance and inflammation. *TheScientificWorldJOURNAL* **2**, 76–88.

---

**This article should be referenced as follows:**

Götte, M. and Echtermeyer, F. (2003) Syndecan-1 as a regulator of chemokine function. *TheScientificWorldJOURNAL* **3**, 1327–1331.

**Handling Editor:**

Edward Goetzl, Principal Editor for *Inflammation* — a domain of *TheScientificWorldJOURNAL*.

---

### BIOSKETCHES

**Martin Götte** obtained his M.Sc. in Biology at the University of Münster, Germany in 1994. After studying the role of Rab GTPases and SNAREs in vesicular trafficking to the yeast vacuole in the laboratory of Dieter Gallwitz at the Max-Planck-Institute of Biophysical Chemistry in Göttingen, Germany, he obtained his Ph.D. in Biochemistry from the University of Göttingen in 1997. From 1997–2000, he studied the role of cell surface heparan sulfate proteoglycans in inflammation, angiogenesis, and tumorigenesis in the laboratory of Merton Bernfield at the Department of Cell Biology of Boston's Harvard Medical School. From 2000–2003, he was a group leader at the Institute of Physiological Chemistry and Pathobiochemistry at Münster University Hospital, Germany, studying syndecans, galactosyltransferases, proteoglycan endocytosis, and basement membrane proteoglycans. In November 2003, he became a faculty member of the Department of Obstetrics and Gynecology of Münster University Hospital, where he continues his work on syndecans. Since 2001, he has been Managing Director of the biotech company Protogeneia, Inc. In 2001, Martin Götte became Prinicpal Editor of the Cell Biology domain, and an Editorial Board Member of the Biochemistry domain of TheScientificWorldJOURNAL.

**Frank Echtermeyer** obtained his M.Sc. in Biology at the Friedrich-Alexander-University of Erlangen-Nuremberg, Germany in 1992. Investigating the function of the newly discovered alpha7 beta1 intergin during migration on different extracellular matrix molecules in the laboratory of Klaus von der Mark at the science groups for rheumatology of the Max-Planck-Society in Erlangen, Germany enabled him to obtain his Ph.D. in Biochemistry from the University of Erlangen-Nuremberg in 1996. From 1996–1999, he studied the role of the heperan sulfate proteoglycan syndecan-4 during wound healing, angiogenesis, and in the formation of focal contacts and actin stress fibers in Paul Goetinck´s group at the Cutaneous Biology Research Centre at Massachusetts General Hospital and Harvard Medical School in Boston. In 1999, he became an independent group leader of the Interdisciplinary Centre for Clinical Science at the Department of Physiological Chemistry and Pathobiochemistry at Muenster University Hospital, Germany. There he continued his work on the role of syndecans during wound healing, development, matrix synthesis, and growth factor mediated signaling. Since 2003, he is a member of the special science area for matrix biology (SFB492) in Muenster.
